# Translation and Psychometric Evaluation in Cancer Care of the German Version of collaboRATE^TM^—a 3‐item Patient‐reported Measure of Shared Decision‐Making

**DOI:** 10.1111/hex.70255

**Published:** 2025-04-03

**Authors:** Pola Hahlweg, Stefan Zeh, Isabelle Scholl, Jördis Zill, Jörg Dirmaier, Paul James Barr, Glyn Elwyn, Martin Härter

**Affiliations:** ^1^ Department of Medical Psychology University Medical Center Hamburg‐Eppendorf Hamburg Germany; ^2^ The Dartmouth Institute for Health Policy & Clinical Practice Geisel School of Medicine, Dartmouth College Lebanon New Hampshire USA; ^3^ Center for Technology and Behavioral Health Geisel School of Medicine, Dartmouth College Lebanon New Hampshire USA

**Keywords:** cultural adaptation, measurement, patient‐reported experience measures, psychometric testing, shared decision‐making, translation

## Abstract

**Introduction:**

The collaboRATE^TM^ measure assesses the shared decision‐making process from patients' perspective with three items. Because of its shortness, it is especially feasible in routine care. It was developed in English and has been translated into several languages. This study aimed to translate collaboRATE into German, test its comprehensibility and evaluate its psychometric properties.

**Methods:**

Translation followed the TRAPD protocol. Comprehensibility was tested in cognitive interviews with lay people (*N* = 18). Psychometric properties were evaluated in a secondary analysis of a sample of 1703 patients with cancer. They rated the collaboRATE items to assess their care experience in general at the respective department of one large university medical centre. We calculated collaboRATE sum and top scores and assessed item characteristics (i.e., acceptance and ceiling effects), convergent validity with the 9‐item Shared Decision‐Making Questionnaire (SDM‐Q‐9 for one specific medical encounter) and satisfaction with care (single item), and divergent validity with psychosocial distress (NCCN distress thermometer). Completion rates, percentages of highest score, skewness, item endorsability and different correlation coefficients informed the evaluation of these psychometric properties.

**Results:**

During translation and cognitive interviewing, the necessity to simplify sentence structures to enhance comprehensibility became apparent. Adaptations led to good comprehensibility. The mean collaboRATE sum score was 82.9 (SD = 19.3), with 466 participants (28.9%) indicating the top score. Item characteristics suggested good acceptability and ceiling effects. Correlations with SDM‐Q‐9 were lower than expected (sum score: *r* = 0.47, *p* < 0.001; top score: pbr = 0.27, *p* < 0.001). Correlations were as expected for satisfaction with care (sum score: *r_s_* = 0.46, *p* < 0.001; top score: *χ* ^2^ = 218.3, *p* < 0.001, Cramer's V = 0.37) and minimally higher than expected for distress (sum score: *r* = −0.11, *p* < 0.001; top score: pbr = −0.09, *p* < 0.001).

**Conclusion:**

A well‐comprehensible German version of collaboRATE is now available. However, ceiling effects were found and convergent validity could not be established in a secondary analysis of a sample from cancer care. Further evaluation is needed regarding the psychometric properties of German collaboRATE.

**Patient or Public Contribution:**

Members of the public were involved in developing the original English collaboRATE and testing the comprehensibility of German collaboRATE. Patients with cancer provided data for psychometric testing.

## 1 Introduction

Shared decision‐making (SDM) has been widely promoted as a central component of high‐quality care within patient‐centred health systems [1]. To accurately assess the current state of SDM and evaluate implementation efforts to foster SDM, psychometrically sound measurement tools are needed [2]. One way to assess uptake of SDM is to ask patients themselves about their experience in questionnaires [3], so‐called patient‐reported outcomes or experience measures (PROMs and PREMs) [4]. To facilitate use in routine care, these should be easy to administer and as brief as possible [5].

The collaboRATE measure is a ‘fast and frugal’ PREM to assess the SDM process from the patient's perspective [6, 7]. The measure was co‐designed with patients through two rounds of cognitive interviews and pilot testing in routine care [6]. It has been used in diverse clinical settings in routine healthcare [8, 9, 10, 11, 12, 13] and is also available as a version for parents or guardians and as a version for individuals acting on behalf of patients (see http://www.glynelwyn.com/collaborate-measure.html). Additionally, collaboRATE is suited to be used in large‐scale patient experience surveys and on the health policy level (e.g., for pay‐for‐performance measurements) due to its brevity and provider group‐level reliability, as has been demonstrated in the United States [14, 15].

CollaboRATE is based on a three‐step SDM model: (1) inform about the health issue, (2) elicit patient preferences and (3) incorporate patient preferences in the decision [6]. The measure uses a formative measurement model [7] and consists of three items: (1) ‘How much effort was made to help you understand your health issues?’; (2) ‘How much effort was made to listen to the things that matter most to you about your health issues?’ and (3) ‘How much effort was made to include what matters most to you in choosing what to do next?’ [6]. The items are preceded by an opening statement (e.g., ‘Thinking about the appointment you have just had…’) and rated on a 10‐point anchored scale ranging from ‘no effort was made’ to ‘every effort was made’ [6]. There are two scoring methods for collaboRATE: sum scores range from 0 to 100, and top scores indicate the percentage of participants that indicated the highest score in all three items [7]. Psychometric testing showed discriminative and convergent validity, inter‐rater reliability and sensitivity to change, but not divergent validity [7].

When standards for translation and cultural adaptation have been met, the availability of the same measurement tools in different languages allows for informed comparisons across studies and countries [16]. CollaboRATE was originally developed in English and has been translated into Arabic, Chinese (Traditional and Simplified Mandarin), Danish, Dutch, French, German (as part of this study), Japanese, Norwegian, Spanish (the United States) and Swedish (see http://www.glynelwyn.com/collaborate-measure.html). Recently, three adapted versions of collaboRATE for paediatric care (i.e., assessed by paediatric patients, parents and parent‐proxy assessment) were developed in German [17]. The authors reported comprehensibility, ‘preliminary face validity’, ‘excellent’ acceptability and ceiling effects [17, 18]. In addition, they found moderate correlations with parental satisfaction with care for the parent version [18].

A German version of the original collaboRATE measure was not available before this study. Thus, the study at hand aimed to translate the original English version of the collaboRATE measure into German, test its comprehensibility and evaluate its psychometric properties.

## 2 Materials and Methods

### 2.1 Study Design

For the translation and psychometric evaluation of collaboRATE, we used a multi‐methods design. Translation of the original English version into German followed the TRAPD protocol (translation, review, adjudication, pre‐testing and documentation) [16]. Comprehensibility of the German version was assessed in cognitive interviews with members of the general public [19, 20]. Regarding psychometric properties of the German version, item characteristics and convergent and divergent validity were assessed using classical test theory [21]. Due to the lack of reporting guidelines for psychometric testing of PREMs, we followed the COSMIN reporting guideline for studies on measurement properties of patient‐reported outcome measures where applicable [22] (cp. Additional file 1).

### 2.2 Phase 1: Translation and Cognitive Interviewing

#### 2.2.1 Translation

The team translation process following the TRAPD protocol [5] consisted of three phases: First, two initial translations were performed by two native German‐speaking healthcare researchers proficient in English and experienced in survey translation P.H. and J.Z.). Second, a third bilingual healthcare researcher, also experienced in survey translation (I.S.), reviewed the two translated versions and suggested a combined version based on the two initial translations. Third, the three researchers (P.H., I.S. and J.Z.) discussed all three versions and derived a consensus version in a review meeting. During the TRAPD translation process, it became apparent that the sentence structure of the first German consensus version (i.e., translated as closely to the English original as possible) was quite complex. Hence, the consensus version was discussed in an additional meeting with four of the authors of this publication with ample experience in survey development and translation (P.H., J.Z., J.D. and M.H.) and a student intern less familiar with the research area. Alternative versions with simplified sentence structure were developed for each item, which were assumed to be more easily comprehensible in German without deviating in content. Both versions were included in the interview guidelines for cognitive testing.

#### 2.2.2 Cognitive Interviewing

We aimed to interview 20 participants from the general public to ensure comprehensibility of the German collaboRATE [19, 20]. We used a combination of convenience and purposive sampling. Notices about the study were distributed in 13 stores and coffee shops throughout the city (community recruitment strategy). Interested parties contacted the study team by phone or email and gave written informed consent before participation. Sampling based on the distribution of gender, age and formal education in Germany was sought. An interview guide was developed within this study, asking patients about the comprehensibility of the items and to rephrase the items in their own words. The guide included the two versions of each item from the translation process: a translation worded as closely as possible to the original English items, and a version as simple as possible in sentence structure. Additional file 2 includes an English translation of the interview guide. Interviews took place in July and August 2017, were audio‐recorded and included a short demographic questionnaire. Interview participants were compensated with 30 Euro. For data analysis, one researcher (S.Z.) collected all comments and suggestions in one document and proposed revisions if applicable. Subsequently, two members of the study team (P.H. and S.Z.) discussed the revisions until a consensus was reached. The resulting prefinal German collaboRATE version was then discussed with the entire study team (S.Z., P.H., J.Z., J.D. and M.H.) and finalised. Descriptive statistics regarding participants' demographic characteristics were calculated using SPSS (IBM SPSS Statistics, version 29).

### 2.3 Phase 2: Psychometric Evaluation

A secondary analysis was performed on a cross‐sectional sample of 2128 patients with cancer from an SDM implementation trial at a comprehensive cancer centre in Germany [23, 24]. As the implementation trial followed a stepped wedge cluster randomised design, each data point could be attributed to either the control condition (i.e., before the department had received the implementation programme) or the intervention condition (i.e., after the department received the implementation programme) [23, 24]. However, the implementation trial did not yield significant differences in patient‐reported SDM between the control and the intervention condition [24].

#### 2.3.1 Participants

Patients were treated at one of three departments of the comprehensive cancer centre: oncology, gynaecology or maxillofacial surgery. Since this is a secondary analysis, inclusion and exclusion criteria were those of the SDM implementation study, the most important being that patients had to be 18 years or older, have sufficient knowledge of the German language and have no severe cognitive impairment [23, 24]. Although the implementation study primarily aimed at patients with cancer, we decided on an inclusive approach for this secondary analysis and included patients with confirmed or suspected malignant neoplasms, in situ neoplasms, neoplasms of uncertain behaviour and benign neoplasms, as long as they were treated at the respective departments. We excluded patients with missing information on diagnosis or other diagnoses than mentioned before. Overall, we decided to use a broad and heterogeneous sample from a cancer care setting for this initial evaluation of the psychometric properties of the German collaboRATE, a generic measure.

#### 2.3.2 Measures

The German collaboRATE was administered in a five‐page survey [23]. In this survey, collaboRATE items were assessed regarding patients' SDM experience in general at the respective department (i.e., not for one specific clinical encounter or decision). The survey also included the 9‐item Shared Decision‐Making Questionnaire [25] (SDM‐Q‐9), a single item on patient satisfaction with care, the German version of the NCCN distress thermometer [26], a single item on the overall health status, and self‐reported questions on demographic and clinical information (e.g., age, gender, education, time since the initial diagnosis, reason for visit and decision topic). The SDM‐Q‐9 is a 9‐item patient‐reported measure, assessing the experienced process of SDM. SDM‐Q‐9 items are rated on a 6‐point Likert scale ranging from ‘completely agree’ to ‘completely disagree’, and sum scores range from 0 to 100. SDM‐Q‐9 items were answered regarding one specific medical encounter at the department. The different framing of collaboRATE and SDM‐Q‐9 was chosen to allow for a more general and a more specific assessment of SDM within the SDM implementation trial. Patient satisfaction was assessed using the single item ‘How satisfied are you with the physician/the treatment team at this hospital in general (i.e., not only regarding your last visit)?’ and answered on a 4‐point Likert scale. The NCCN distress thermometer measures the patient's distress over the last week with a single item on an 11‐point scale.

#### 2.3.3 Data Collection

During four measurement waves of the SDM implementation trial between March 2018 and August 2020, patients were approached in the waiting areas and in the inpatient wards of the respective departments and asked to fill out the anonymous paper‐and‐pencil survey. As the SDM implementation trial sought effects on the department level using a broad understanding of healthcare decisions, patients were not excluded depending on the reason for their visit or the decision topic. If feasible, a clinical encounter that took place immediately before filling out the survey should be assessed, and the questionnaire should be handed in before leaving the hospital. If not feasible, participants could assess a different clinical encounter, indicate the date of the encounter on the questionnaire and were offered to take the survey with them and return it by mail. All participants gave written informed consent before participation. Data entry included a blinded double entry of 10% of data for quality control.

#### 2.3.4 Data Analysis

We allowed for half‐point ratings in the few instances those occurred (*n* = 6 for Item 1, *n* = 7 for Item 2 and *n* = 5 for Item 3), and missing values were not imputed. Sensitivity analyses were calculated for the control condition only, the intervention condition only and each of the three departments separately. For the German collaboRATE, sum scores (ranging from 0 to 100) and top scores (i.e., percentage of respondents indicating the highest rating in all items) were calculated. Acceptability was operationalized through missing data frequencies of less than 10% and corresponding completion rates [4, 27]. Item endorsability (also called item difficulty) as a psychometric property of floor and ceiling effects was assessed by dividing item means by the maximum value on the 10‐point anchored scale ranging from 0 to 9, aiming at item difficulties between 0.2 and 0.8 [28]. Furthermore, floor or ceiling effects were assumed if more than 15% of participants chose the lowest or highest score [29]. For these item characteristics, we calculated additional sensitivity analyses for partial samples allocated by age, gender, education level and setting. To test for convergent and divergent validity, we calculated Pearson product‐moment or Spearman rank correlation coefficients with collaboRATE sum scores and point‐biserial correlation coefficients or *χ* ^2^ tests and Cramer's V with collaboRATE top scores. Table 1 shows the levels of measurement for each outcome and the correlation coefficient was used for each test.

**Table 1:** Tests used to assess convergent and divergent validity.

	collaboRATE sum score (interval)	collaboRATE top score (nominal)
SDM‐Q‐9 sum score (interval)	Pearson product‐moment correlation coefficient	Point‐biserial correlation coefficient
Patient satisfaction with care (ordinal)	Spearman rank correlation coefficient	*χ* ^2^ test and Cramer's V
Patient distress (interval)	Pearson product‐moment correlation coefficient	Point‐biserial correlation coefficient

*Note:* The level of measurement for each outcome is shown in brackets and informed the choice of the specific correlation coefficient (e.g., interval × interval = Pearson product‐moment correlation coefficient; interval × ordinal = Spearman rank correlation coefficient).

Corresponding to prior research [7, 13, 18, 30], we expected correlations of at least 0.7 with the SDM‐Q‐9 and 0.5 with patient satisfaction with care. In terms of divergent validity, correlations of 0.1 with patients' distress were hypothesised. Theoretical considerations and existing literature about SDM, patient satisfaction and patient distress informed these thresholds. As patient satisfaction with care has been found to be associated with SDM [31, 32], but is conceptually not the same, we expected moderate correlations between collaboRATE and patient satisfaction. Patient distress, on the other hand, had been found to not be associated with SDM [33]. The study team agreed upon all thresholds before data analysis. Due to the formative measurement model, internal consistency was not assessed. The study design did not allow assessing retest reliability. Due to the lack of differences in SDM between the implementation and the control condition, we were not able to assess discriminative validity and sensitivity to change in this sample. Data were analysed using SPSS (IBM SPSS Statistics, version 29).

#### 2.3.5 Power Analyses

As this is a secondary analysis, the a priori sample size calculation for the dataset was undertaken for the SDM implementation trial and described elsewhere [23, 24]. Post hoc power analyses were performed with G*Power for the six tests assessing convergent and divergent validity, and power was revealed to be equal to or greater than 0.95 for all analyses. The alpha error probability was set to 0.05, and sample sizes ranged between *n* = 1429 and *n* = 1590 for the different tests.

## 3 Results

### 3.1 Phase 1: Cognitive Interviewing

We conducted 18 cognitive interviews with a mean duration of 20 min (SD = 4.2, range 12–26 min). Two participants missed their scheduled interview, which led to 18 instead of 20 interviews. The demographic characteristics of the interview participants are displayed in Table 2. Sampling criteria could only be approximated with fewer participants older than 60 years, fewer male participants and a slightly different distribution of education levels (see Additional file 3). Cognitive interviews showed that the original item versions (i.e., translated as closely to the English originals as possible) were less comprehensible than the versions with simplified sentence structure. Hence, we chose the adapted versions for the final German collaboRATE, for which we found good comprehensibility. Consequently, the rating scale had to be adapted to a scale ranging from ‘not at all’ to ‘very much’ (instead of ‘no effort was made’ to ‘every effort was made’). The final German items (translated back to English) were

1) ‘How much were you helped to understand your health issues?’
2) ‘Regarding your health issues: How much were you listened to about what matters most to you?’
3) ‘When choosing what to do next: How much has been considered what matters most to you?’

Additional file 4 includes the final German collaboRATE measure.

**Table 2:** Demographic characteristics of participants of cognitive interviews (*N* = 18).

Age in years, mean (SD) [min; max]	45.3	(16.5)
		[20;75]
Gender, *n* (%)		
Female	11	(64.7)
Male	6	(35.3)
Formal education, *n* (%)		
Low^a^	1	(5.6)
Intermediate^b^	5	(27.8)
High^c^	7	(38.9)
Very high^d^	5	(27.8)
Occupational status, *n* (%)		
(Self‐)employed, full time	7	(38.9)
Retired/permanently unable to work	5	(27.8)
Student	4	(22.2)
Sick leave	1	(5.6)
Unemployed	1	(5.6)
Mother tongue, *n* (%)		
German	16	(88.9)
Other	2	(11.1)
Proficiency in German, mean (SD) [min; max]	9.1	(1.7)
1 to 10, higher better		[3; 10]

*Note:* Frequencies not adding up to the total number of participants within groups indicate missing data; percentages are calculated for valid data within the group.

Abbreviations: min, minimum; max, maximum; *n*, number; SD, standard deviation.

^a^ Low = no formal degree or graduation after less than 10 years at school.

^b^ Intermediate = graduation after 10 or 11 years at school.

^c^ High = graduation after more than 11 years at school.

^d^ Very high = college or university degree.

### 3.2 Phase 2: Psychometric Evaluation

#### 3.2.1 Response Rate and Sample Characteristics

2131 analysable patient surveys were returned out of 4224 invited patients (50.4%). Non‐participation in the survey was most frequently explained by prior participation in the survey (*n* = 459), physical or psychological burden (*n* = 225) or no interest in the study (*n* = 183). 809 patients did not give a reason for non‐participation. We furthermore excluded participants not meeting inclusion criteria (i.e., being younger than 18 years, *n* = 2; not having indicated an oncological or related diagnosis, *n* = 426), leaving us with a dataset of *n* = 1703 questionnaires from patients with heterogeneous demographic and clinical characteristics (such as time since diagnosis, reason for visit and decision topic). In this dataset, almost two‐thirds of the sample were female (60.9%), and the mean age was 58 years, with a range from 18 to 97 years. Most patients had a confirmed cancer diagnosis (93.2%). Table 3 gives a detailed overview of patient characteristics within the surveyed sample. Additional file 5 includes sample descriptions for all partial datasets used in the sensitivity analyses.

**Table 3:** Demographic characteristics of the sample for psychometric testing (*N* = 1703).

Age in years, mean (SD) [min; max]	58.0	(15.2)
		[18; 97]
Gender, *n* (%)		
Female	1027	(60.9)
Male	658	(39.0)
Other or not specified	1	(0.1)
Formal education, *n* (%)		
Low^a^	295	(17.7)
Intermediate^b^	518	(31.1)
High^c^	381	(22.9)
Very high^d^	458	(27.5)
Other	14	(0.8)
Occupational status^e^, *n* (%)		
Retired	756	(45.1)
(Self‐)employed	741	(44.2)
Other^f^ (< 5% each)	255	(15.2)
Mother tongue, *n* (%)		
German	1525	(92.9)
Other	107	(6.5)
Bilingual (German and other)	10	(0.6)
Setting, *n* (%)		
Outpatient	1548	(91.1)
Inpatient	152	(8.9)
Diagnosis, *n* (%)		
Confirmed malignant neoplasm	1588	(93.2)
Suspected malignant neoplasm	20	(1.2)
In situ neoplasm or neoplasm of uncertain behaviour	54	(3.2)
Benign neoplasm	41	(2.4)
Time since the initial diagnosis, *n* (%)		
1 year or less	671	(43.8)
1 to 5 years	567	(37.0)
More than 5 years	271	(17.7)
Reason for visit^e^, ^g^, *n* (%)		
Diagnostic investigation	301	(17.9)
Initial communication of the diagnosis	270	(16.1)
Treatment planning	437	(21.1)
Treatment	347	(20.7)
Treatment monitoring	503	(30.0)
Aftercare	364	(21.7)
Decision topic^e^, ^g^, *n* (%)		
Diagnostic procedures	474	(29.5)
Surgery	454	(28.3)
Chemotherapy	527	(32.8)
Radiation therapy	149	(9.3)
Other treatment	169	(10.5)
Overall health, mean (SD) [min; max]	3.2	(0.8)
1 to 5, higher better		[1; 5]
Distress, mean (SD) [min; max]	5.6	(2.4)
0 to 10, higher more		[0; 10]
SDM‐Q‐9 sum score, mean (SD) [min; max]	64.4	(26.5)
1 to 100, higher more SDM		[0; 100]
Patient satisfaction, mean (SD) [min; max]	3.5	(0.8)
1 to 4, higher more		[1; 4]

*Note:* Frequencies not adding up to the total number of participants within groups indicate missing data. Percentages are calculated for valid data within the group.

Abbreviation: SD = standard deviation.

^a^ Low = no formal degree or graduation after less than 10 years at school.

^b^ Intermediate = graduation after 10 or 11 years at school.

^c^ High = graduation after more than 11 years at school.

^d^ Very high = college or university degree.

^e^ Multiple choices are possible.

^f^ Including homemaker, student/trainee, sick leave, parental leave, military service and unemployed.

^g^ Only categories with more than 5% of the total sample are displayed.

#### 3.2.2 Sum Score and Top Score of German collaboRATE

**Figure 1:**
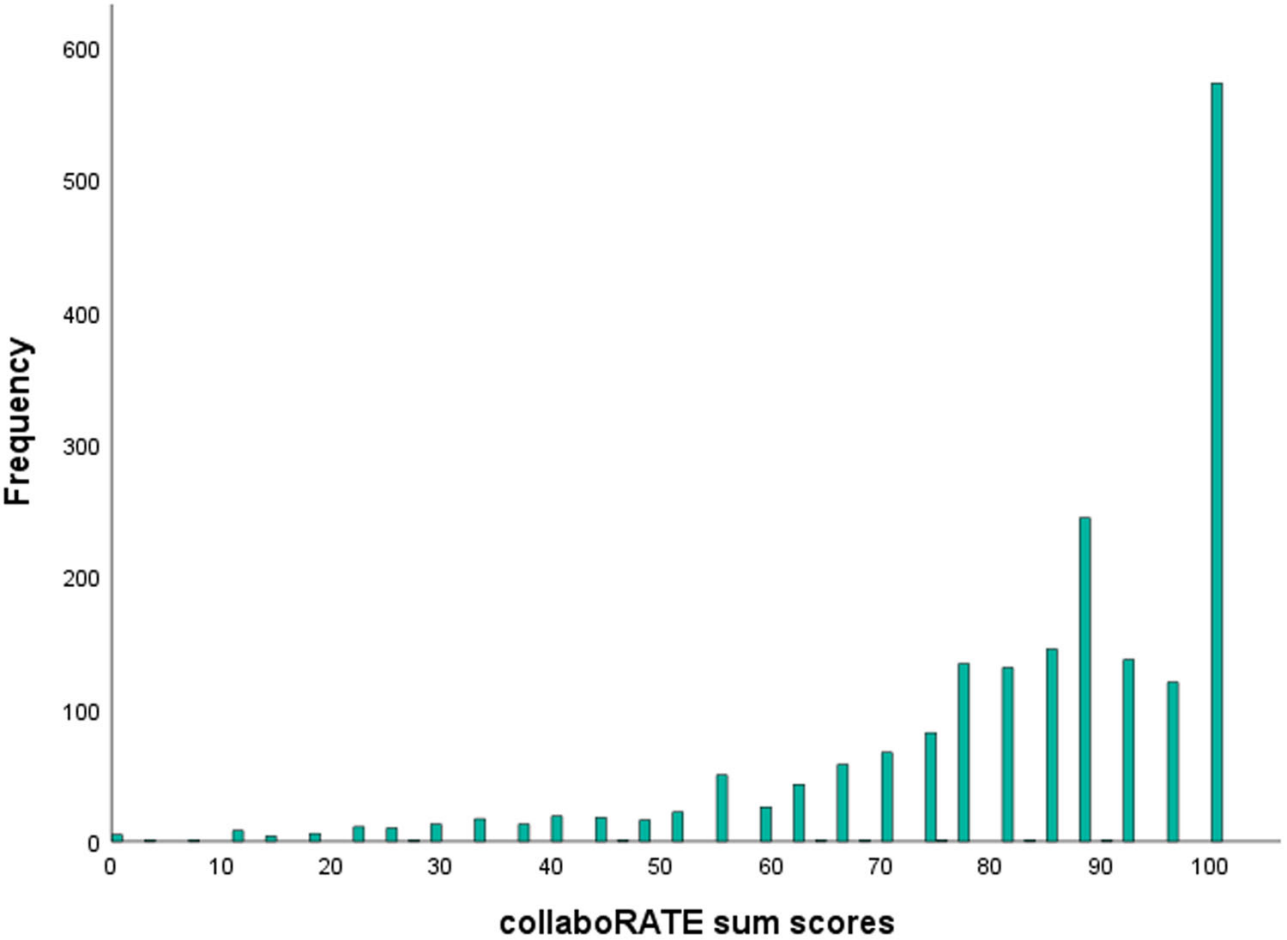
Frequency distribution of collaboRATE sum scores.

We calculated collaboRATE sum scores and top scores for *n* = 1610 questionnaires (i.e., questionnaires with valid responses in all three collaboRATE items). The mean collaboRATE sum score was 82.89 (SD = 19.32, range 0–100). Frequency distributions of collaboRATE sum scores are shown in Figure 1. The collaboRATE top score was 28.9% (*n* = 466). The results of the sensitivity analyses with partial datasets can be found in Table 4.

**Table 4:** Sum score and top score of the German collaboRATE.

		collaboRATE sum score (range 0–100)		collaboRATE top score	
	*n*	Mean	SD	*n*	%
Complete sample	1610	82.89	19.32	466	28.9
Partial samples					
Control condition	773	82.89	19.89	244	31.3
Intervention condition	834	82.93	18.72	223	26.7
Dept. of Oncology	724	81.88	19.10	180	24.9
Dept. of Maxillofacial Surgery	310	82.38	20.65	92	29.7
Dept. of Gynecology	573	84.51	18.66	193	33.7

*Note:* Percentages are calculated for valid data within the group.

Abbreviations: Dept, = Department; SD = standard deviation.

#### 3.2.3 Missing Data and Acceptability

Out of 5109 possible collaboRATE data points (1703 surveys × 3 items), 170 were missing (3.3%). On the item level, 45 (2.6%), 48 (2.8%) and 77 (4.5%) missing values occurred for Items 1, 2 and 3, respectively. Accordingly, item completion rates were 97.4%, 97.2% and 95.5%. Out of 1703 surveys, 1610 had valid responses in all three collaboRATE items (94.5%), 51 had missing in one item (3.0%), 7 in two items (0.4%) and 35 in all three items (2.1%).

#### 3.2.4 Item Characteristics

Response distributions of each collaboRATE item are presented in Additional file 6. Item characteristics are included in Table 5. We found completion rates between 97.4% and 95.5%, indicating acceptability. Item endorsability was above the threshold of 0.8, suggesting ceiling effects. This aligns with more than 15%, indicating the highest score for all items and the negative skewness of the items. Results of sensitivity analyses with different partial samples can be found in Additional file 7. The exploratory descriptive analyses of different partial samples did not suggest major differences between the partial samples.

**Table 5:** Item characteristics.

Item	*n*_valid_	Mean	SD	[min; max]	Skewness	Item difficulty	Completion rate, %	‘Not at all’, %^a^	‘Very much’, %^a^
Item 1	1658	7.54	1.79	[0; 9]	−1.64	0.84	97.4	0.5	39.4
Item 2	1655	7.51	1.84	[0; 9]	−1.65	0.83	97.2	0.6	39.3
Item 3	1626	7.32	2.10	[0; 9]	−1.63	0.81	95.5	1.7	37.1

*Note:* All items were rated on a 10‐point anchored scale ranging from 0 (‘not at all’) to 9 (‘very much’); Item 1 = ‘How much were you helped to understand your health issues?’; Item 2 = ‘Regarding your health issues: How much were you listened to about what matters most to you?’; Item 3 = ‘When choosing what to do next: How much has been considered what matters most to you?’.

Abbreviation: SD = standard deviation.

^a^ Percentages were calculated for valid data for this item.

#### 3.2.5 Convergent and Divergent Validity

When comparing German collaboRATE scores with the SDM‐Q‐9, we found a Pearson product‐moment correlation of *r* = 0.47 for the collaboRATE sum score and a point‐biserial correlation of pbr = 0.27 for the collaboRATE top score (both *p* < 0.001). Both were lower than the hypothesised correlation of 0.7 to show convergent validity. The Spearman rank correlation between collaboRATE sum scores and satisfaction with care was as hypothesised (*r_s_* = 0.46, *p* < 0.001). When comparing collaboRATE top scores with satisfaction, Cramer's V was 0.37 (*χ* ^2^ = 218.30, *p* < 0.001), below the expected 0.5. The correlation between collaboRATE and distress matched our hypotheses (*r* = −0.11 for sum score, *p* < 0.001; pbr = −0.09 for top score, *p* < 0.001). Results of the sensitivity analyses can be found in Additional file 8.

## 4 Discussion

This paper adds a German translation of the collaboRATE measure assessing patient‐reported SDM with three items. Cognitive interviews yielded the need to simplify sentence structure, leading to a comprehensible German version of collaboRATE. The German collaboRATE version was psychometrically tested in cancer care as a secondary analysis of data from a large‐scale SDM implementation trial. Psychometric testing indicated good acceptability, relatively high scores in item endorsability and ceiling effects in all three items. The correlations assessing convergent validity with another SDM measure were lower than expected and with satisfaction with care were partially as expected. Correlations with psychosocial distress assessing divergent validity were as expected.

The good results on comprehensibility and acceptability of the German collaboRATE make it a highly feasible measure for use in routine care and other contexts with limited time and resources. During translation and cognitive testing, we discovered the need to modify sentence structure between the original English and the German versions to ensure comprehensibility. These changes were grammatical changes due to linguistic differences between English and German and are assumed not to have changed the meaning of the items. This is comparable to experiences made with a paediatric German version [17]. Cognitive interviews with Spanish‐speaking populations also showed a need for adaptation [34, 35].

Ceiling effects are a major challenge to patient‐reported measures in general [36] and to collaboRATE in particular. The developers of the measure, therefore, suggested using top score percentages instead of means for collaboRATE [7]. In our sample, we also found ceiling effects in all three items demonstrated by negative skewness, high item endorsability and high percentages in choosing the best score. These ceiling effects could point to limited variability within the scores, which can limit a measure's ability to differentiate between more and less SDM. Thus, future studies should evaluate collaboRATE's discriminative validity and sensitivity to change, preferably in routine care. So far, we only know of one study that has done so in simulated encounters and demonstrated discriminative validity as well as sensitivity to change [7]. If the German collaboRATE should be found to have difficulty detecting differences in patient experiences, consequences such as using the top score assessment or recalibrating the scale could be considered in the future. Prior studies on PREMs suggested that top score assessment might be a way to better reflect the true meaning of the values and more accurately detect changes [36]. If limited discriminative validity and sensitivity to change of collaboRATE were established, they may have contributed to the lack of effects found in the implementation trial with collaboRATE as an outcome.

All comparisons between our results and previous studies have to bear in mind the different framing of the assessments (i.e., for one specific encounter or for the overall experience in this department). When comparing our results to those of other international studies using collaboRATE, top scores in our sample were lower than in several US samples (28.9% in our sample, compared to approximately 60% to 80% in different US samples) [9, 13, 15, 37]. One US‐based study including surgery patients found similar collaboRATE top scores as we did [12], and two studies from Europe (i.e., mental health in Spain and diverse patient population from different medical specialities and hospitals in the Netherlands) reported only slightly higher top scores (39.8% and 37.5%) [10, 30]. The two studies from Europe [10, 30] also reported similar collaboRATE sum scores as in our sample. Many other studies refrained from reporting sum scores. Ceiling effects were seldom discussed, but if our study with relatively low collaboRATE sum and top scores found ceiling effects, it is likely that ceiling effects were present in other samples as well. In general, possible effects of differences in questionnaire responses between cultures on collaboRATE scores should be considered, before collaboRATE in different languages will be used to assess SDM cross‐culturally [38].

Regarding convergent validity with another well‐established SDM measure [25], we found lower correlations than expected. This is likely influenced by the fact that we used different frames for the two measures in this study: While collaboRATE was assessed for patients' SDM experience in general at the respective department, SDM‐Q‐9 was assessed for their experiences in one specific medical encounter. The fact that we found moderate correlations despite the different frames could indicate a limited influence of the framing on the scores and should be further explored. Nevertheless, these correlations are similar to those found in the US surgical sample [12] and the Dutch sample [30], but lower than in the original collaboRATE validation [7]. To tackle this issue, we suggest, as a first step, re‐testing convergent validity between the German collaboRATE and SDM‐Q‐9 with adjusted frames and an additional SDM measure for comparison. However, weak correlations between different measures of SDM are a known phenomenon. For example, correlations of 0.19 between the patient‐reported and the physician‐reported SDM‐Q‐9 [39] and correlations of 0.29 between the patient‐reported SDM‐Q‐9 and another measure of patient‐perceived involvement in decision‐making were reported [40]. However, correlations of 0.70, 0.83 and 0.86 were found between an adapted SDM‐Q‐9 version for psychiatry and three other SDM‐related endpoints in another study [41]. The heterogeneity in results on whether different SDM‐Q‐9 measures assess the same construct points towards an overarching need to improve SDM conceptualisation and measurement further.

Regarding patient satisfaction with care, correlations with collaboRATE scores between approximately 0.4 and 0.6 have consistently been found [13, 18]. We argue that the degree to which SDM measures (should or should not) assess satisfaction with care needs further theoretical and empirical clarification. If one wants to develop the measurement of different constructs of patient experience in healthcare further, establishing sound models that describe their interconnectedness could be a valuable next step.

Whether the psychometric properties of collaboRATE are influenced by the framing of the items (i.e., assessment for one specific encounter or for the overall experience in this department) remains an open question. As described above, our results using the latter framing seem to be comparable to previous studies using the frame of one specific encounter. Nevertheless, subsuming a range of experiences into one score might be challenging for participants. On the other hand, in a study assessing the response process validity of SDM‐Q‐9, participants reported difficulty assessing one specific encounter only instead of their overall experience [42]. As both collaboRATE and SDM‐Q‐9 are PREMs assessing SDM, this might be true for collaboRATE as well.

### 4.1 Strengths and Limitations

The combination of thorough translation, cognitive interviewing with participants from the general public and psychometric evaluation in a large clinical sample of cancer patients is a major strength of this study. The clinical sample came from routine care in different medical specialities in a university medical centre setting. To our knowledge, no psychometric evaluation of collaboRATE has been undertaken in a cancer sample before this study. As our sample included patients with different education levels, a variety of different cancer diagnoses, variable time since initial diagnosis, different reasons for their visit and decisions to be made, we believe that generalisability across German cancer patients is likely even though all patients were treated in one comprehensive cancer centre in Germany. However, psychometric properties of collaboRATE in other settings and conditions need to be established in future studies [21]. For example, we recommend complementing our broad sample with samples of patients facing specific healthcare decisions. Furthermore, this study, being a secondary analysis of data from an SDM implementation trial, limited the psychometric evaluation. CollaboRATE was assessed for the general SDM experience at the respective department instead of for one encounter and as part of a five‐page survey. This limited comparability with other studies and the assessment of convergent validity and made survey fatigue and ordering effects possible. Selection and non‐response biases are also a possibility, even though we made an effort to include patients free of bias in the primary implementation trial. In addition, a more differentiated evaluation of collaboRATE in in‐ and outpatient settings would be beneficial in the future. Lastly, we were not able to assess discriminative validity and sensitivity to change in this sample. We recommend testing these two criteria in clinical samples in the future. We also recommend exploring if and how item‐response theory could enrich further testing of collaboRATE that uses a formative measurement model.

## 5 Conclusion

A German version of collaboRATE is now available. This measure has potential, as it is highly feasible due to brevity, comprehensibility and acceptability. This study was one of the first steps towards adding a German collaboRATE version to the already existing translations and enabling international comparisons of patients' experienced level of SDM. However, further exploration of construct validity is needed, especially with different framing, in additional settings and samples, and in terms of convergent validity, discriminative validity and sensitivity to change. This measure could then be on the way to becoming one of the best questionnaires for assessing SDM.

## Author Contributions

**Pola Hahlweg:** conceptualisation, supervision, methodology, project administration, writing – original draft, investigation, formal analysis, data curation, writing – review and editing, visualisation, funding acquisition. **Stefan Zeh:** methodology, formal analysis, writing – review and editing, investigation. **Isabelle Scholl:** conceptualisation, supervision, writing – review and editing, data curation, funding acquisition. **Jördis Zill:** conceptualisation, supervision, methodology, investigation. **Jörg Dirmaier:** conceptualisation, supervision, investigation, writing – review and editing. **Paul James Barr:** conceptualisation, supervision, writing – review and editing, resources. **Glyn Elwyn:** conceptualisation, writing – review and editing, supervision, resources. **Martin Härter:** conceptualisation, supervision, investigation, writing – review and editing.

## Ethics Statement

The PREPARED trial contributed the data for the psychometric evaluation in this publication and was approved by the Ethics Committee of the Medical Association Hamburg, Germany (study ID: PV5368). For the qualitative cognitive interviews to test comprehensibility with members from the public, we sought ethical approval from the Ethics Committee of the Medical Association Hamburg, Germany, and received the information that a medical ethics board approval was not applicable in this case (email correspondence from March/April 2017, no study ID assigned). All phases of this study were conducted in accordance with the latest version of the Helsinki Declaration of the World Medical Association. Principles of good clinical practice were respected, and requirements of data protection were met.

## Consent

Study participation was voluntary. Potential participants received oral and written information and had the possibility to ask further questions. We obtained written informed consent from all participants of both phases of the study.

## Conflicts of Interest

P.H., S.Z., I.S., J.Z., J.D., P.B., G.E. and M.H. declare that the research was conducted in the absence of any commercial or financial relationships that could be construed as a potential conflicts of interest.

## Supporting information

Hahlweg et al collaborate German supporting material.

## Data Availability

The qualitative data collected and analysed during the translation process (in German) are available from the corresponding author upon reasonable request. Additional restrictions apply to the dataset used for psychometric evaluation (secondary analysis). De‐identified data that support the findings of this study are available upon reasonable request. Investigators who propose to use the data have to provide a methodologically sound proposal directed to the corresponding author (Dr. Pola Hahlweg) and the principal investigator of the main study (Dr. Isabelle Scholl). For both datasets, signing a data use/sharing agreement will be necessary, and data security regulations both in Germany and in the country of the investigator who proposes to use the data must be complied with. Preparing datasets for use by other investigators requires substantial work and is thus linked to available or provided resources.
