# Supplementary material for: Translation and Psychometric Evaluation in Cancer Care of the German Version of collaboRATETM—a 3‐item Patient‐reported Measure of Shared Decision‐Making
Source: Health Expect. 2025 Apr 3;28(2):e70255. doi: 10.1111/hex.70255 (PMC11968779; doi:10.1111/hex.70255)
Supplement: Supplementary file 1 — Hahlweg et al collaborate German supporting material. [file HEX-28-e70255-s001.pdf]

Supplementary material

- Additional file 1: COSMIN reporting guideline
- Additional file 2: Interview guide for cognitive interviews
- Additional file 3: Stratification of participants of cognitive interviews
- Additional file 4: Final German collaboRATE measure
- Additional file 5: Demographic characteristics of the main sample and data sub-sets used for sensitivity analyses
- Additional file 6: Response distributions of the German collaboRATE items
- Additional file 7: Item characteristics in the main sample and data sub-sets used for sensitivity analyses
- Additional file 8: Convergent and divergent validity in the main sample and data sub-sets used for sensitivity analyses

COSMIN reporting guideline

From Gagnier JJ, Lai J, Mokkink LB, Terwee CB. COSMIN reporting guideline for studies on measurement properties of patient-reported outcome measures. *Qual Life Res*. 2021;30(8):2197-2218. doi:10.1007/S11136-021-02822-4

**General reporting recommendations relevant for all studies on measurement properties**			
Item no.	***Item name***	***Item description***	***Page***
Report section: title			
T1	Patient-reported outcome measure (PROM)	The name of the PROM instrument(s) (and version if relevant) being studied	1
T2	Measurement property (MP)	What MPs are being studied or more generally, that MPs are being studied (if there are many properties being investigated, for example)	1
T3	Study sample	General description of relevant study sample characteristics (e.g., condition of interest, language) and also any intervention or exposure (e.g., treatments) if applicable	1
Report section: abstract			
A1	PROM	The name of the PROM instrument(s) (and version if relevant) being studied (i.e., the SF-36 or SF-12; language version) or if it concerns an item bank (e.g., PROMIS instruments). The type of instrument (e.g., a self-reported questionnaire or interview)	2
A2	Measurement property	What MPs are being studied or more generally, that MPs are being studied (if there are many properties being investigated, for example)	2
A3	Design	The type of study being used to test the properties (e.g., test–retest design, longitudinal study, cohort, cross sectional, case series, randomized etc.). Other details of the study design if relevant (intervention/exposure, description of comparison instruments, outcomes other than PROMs)	2
A4	Sample	Inclusion / exclusion criteria. General description of relevant study sample characteristics (e.g., condition of interest, geographic location, language, other relevant demographic and baseline characteristics)	2
A5	Methods	A brief description of the methods for investigating each MP including statistical analyses	2
A6	Results	The main results for all MPs investigated reporting statistics for each result with measures of precision where appropriate	2
A7	Discussion/ Conclusions	A brief description of the results in the context of existing evidence, main strengths and drawbacks and the need for future research on the PROM(s) investigated	2
Report section: introduction			
I1	Name and describe the PROM of interest	Specify the name, type, language, and version of the PROM being investigated and how it was developed. Describe the construct the PROM aims to measure and its subscales; describe the structure of the PROM (e.g., the number of factors, the number of items, scoring algorithm); describe relevant instructions (like time period), and number or type of response categories. State whether the PROM is based on a reflective or formative model<br>Note: This information may also appear in the methods section in greater detail	4-5
I2	Target population	Describe the specific target population that the PROM was designed for. The authors need to provide the appropriate and necessary characteristics of this population	4
I3	Citation for the original development of the PROM	The citation for the original development paper(s) should be provided and other highly relevant citations related to the quality of the specific PROM under investigation	4

I4	State of knowledge & Rationale	A description of the current scientific knowledge (what is known) regarding the MPs of? the PROM under investigation. The authors should provide a literature review or refer to a recent review of all existing evidence of the specific version (e.g., language, short form) of the PROM and explain why the new study is necessary and important. The rational for the current proposed study should be given	4-5
I5	Definitions	Specialized terms should be defined or explained	n/a
I6	Objectives and hypotheses	State the specific objective(s) of the research and hypotheses related to the specific PROM under investigation	5
Report section: general methods			
GM1	Study design	State the key elements of the study design	5
GM2	Participants	State how the participants were chosen; the inclusion and exclusion criteria. (e.g., if a PROM for a specific condition, then the eligibility and selection criteria should reflect this)	6
GM3	PROM administration	An explicit description of how and when the PROM(s) were administered (e.g., in what setting) including data collection devices/system used (e.g., paper-based, electronic administration / ePRO) should be provided	7
GM4	Data collection procedures	Provide information about other data collection, exposure methods (e.g., allocation to interventions) and time points / follow-up points	7
GM5	Power/sample size calculation	Provide a power calculation for all MP analyses. Alternatively, if a rule of thumb is used, state it and the source/citation	n/a
GM6	Statistical analyses	Statistical analyses and tests corresponding to all hypotheses or objectives for all MPs should be reported. Where appropriate, a cutoff for statistical significance should be reported (e.g., p-value less than 0.05). A description of all statistics to be used to estimate the magnitude and direction of effect should also be reported, together with measures of variability or precision. Report statistical package used	7-8
GM7	Missing data	State approaches or plan for dealing with missing data	7
GM8	Post hoc analysis	The report should specify analyses that used data after the data collection period concluded (i.e., if the analyses were post hoc; secondary data analyses) and describe the rationale for any post hoc analyses	6
Report section: general results			
GR1	Missing data	The amount and reasons for missing data should be explained for all analyses for all PROMs (or other outcome measurement instruments) and relevant groups	10, 12
GR2	Participant/patient Characteristics	The study patients' characteristics should be described, including baseline PROM scores	10-11
GR3	Sample size	If one study contained analyses using different sample sizes, the authors should report the sample size for each analysis	10
Report section: discussion			
D1	MP evidence	Per measurement property the authors should compare the result to the criteria for good measurement properties (e.g., COSMIN criteria) [25], and determine if the specific MP is sufficient or not. Note: This information may also appear in the results section in greater detail in a table for example	14
D2	Practical relevance	The authors need to discuss the practical relevance of the findings	14-16
D3	Strengths and limitations	Strengths and limitations of the study should be discussed. For example, discuss if there were any significant potential biases in the study that could have impacted the results	16
D4	Generalizability	Generalizability issues related to the PROM results should be discussed. For example, discuss if the results could be generalized to other populations given the sample studied	16

D5	Instrument changes	Discuss the need for modifications to the existing PROM or new PROM development. If you conclude that one of the measurement properties is insufficient, you could suggest some modification, or if it is really poor, you could suggest stopping use of the PROM (in the specific population or in general)	14-16
D6	Future research	Report specifically the type of research needed to answer new questions arising out of these findings for the particular MP and PROM investigated	14-16
Report section: conclusions			
C1	Conclusions	State the overall conclusions for each MP and of the use PROM investigated	16
Report section: other information			
O1	Conflict of interest	State any relevant conflict of interest related to the PROM under investigation (e.g., an author being the PROM developer, funding body etc.)	17
Specific reporting recommendations for studies on content validity			
CV1	Relevance	Report if and how patients and/or professionals were asked whether each item is relevant for their experience with the condition	n/a
CV2	Comprehensiveness	Report if and how patients and/or professionals were asked whether all key concepts are included	n/a
CV3	Comprehensibility	Report if and how the comprehensibility of the PROM instructions, items, response options, and recall period was assessed	6, 8
CV4	Relevance results	Report if all items were considered relevant for the construct, population, and context of use of interest by patients and/or professionals	n/a
CV5	Response options and recall period	Report whether the response options and recall period were considered appropriate by patients and/or professionals	8
CV6	Comprehensiveness results	Report whether patients and/or professionals considered all key concepts to be included in the PROM	n/a
CV7	Comprehensibility results	Report whether patients understood the PROM instructions, items, and response options as intended	6, 8
Specific reporting recommendations for studies on structural validity			
SV1	Factor analyses: classical test theory (CTT) PROMs	Report details of the methods and results for any exploratory or confirmatory factor analyses. State the rational for any explorative factor analyses (e.g., no clear a priori hypotheses). For CFA, describe and justify the factor structure of tested models. Methods and results for checking of the assumptions should be described, the method of estimation, goodness-of-fit statistics and cutoff points for good model fit, including factor loadings of best-fitting model	n/a
SV2	Item Response theory (IRT) analyses	Type of IRT/Rasch model should be reported. Also report the method of estimation, methods and results for checking of the assumptions (unidimensionality (see factor analysis), local dependency (e.g., residual correlations), monotonicity; (e.g., Mokken scaling), goodness-of-fit statistics, and cutoff points for goodness of item/model fit, and all item parameters	n/a
Specific reporting recommendations for studies on internal consistency			
IC1	Unit of measurement	Report internal consistency methods and results for each unidimensional scale or subscale. Report all evidence or assumptions associated with unidimensionality	n/a
IC2	Continuous scores	Report Cronbach's alpha or omega statistics. Report other statistics calculated for internal consistency of continuous scores	n/a
IC3	Dichotomous scores	Report Cronbach's alpha or Kuder–Richardson coefficient. Report other statistics calculated for internal consistency of dichotomous scores	n/a
Specific reporting recommendations for studies on cross-cultural validity\measurement invariance			
CCV 1	Comparator group(s)	Report characteristics of (sub)groups being compared. Include sample sizes in each group	n/a

CCV 2	Factor analyses: classical test theory (CTT) PROMs	Report details of the methods and results for multiple-group confirmatory factor analyses, logistic regression analyses, or other analyses performed. Describe and justify the series of tested models, including constraints of factor loadings, intercepts and variances in CFA. Methods and results for checking of the assumptions should be described. Criteria to define invariance. Describe the method of estimation, goodness-of-fit statistics and criteria used to flag items for measurement invariance	n/a
CCV 3	Item response theory (IRT) analyses	Type of IRT/Rasch model should be reported. Also report the methods and results for checking of the assumptions (unidimensionality (see factor analysis), local dependency (e.g., residual correlations), monotonicity; (e.g., Mokken scaling). Describe statistical packages, method of estimation, criteria used to flag items for DIF, and methods and results of all model comparisons	n/a
Specific reporting recommendations for studies on reliability			
R1	PROM administrations	Report the total number of measurements made and if the measurements were applied to the same samples using the same PROM. The process of administrating the measurements to the patients should be described, including who administered it (i.e., did the patient complete it or was there a proxy), when, how and any time intervals between administrations should be reported. This should include: time interval between repeated measurements (e.g., was the patient stable or not), the test type (e.g., a self-administered questionnaire, an interview-based PROM), the setting in which the instrument was administered (e.g., at the hospital, or at home), and the instructions given for completing it. If relevant, other instruments or measurements accompanying the repeated PROM measurement. Also, if relevant, the independence (whether the PROM was completed without knowledge of the previous scores) of the administrations	n/a
R2	Statistical analyses	All statistical analyses and results specific to the reliability assessment(s) should be described and their use justified (e.g., the intraclass correlation coefficient (ICC) model or type of Kappa coefficient used). Also, describe the variance components, and the weighting scheme used for ordinal scores (e.g., linear or quadratic weights)	n/a
R3	Methods to improve reliability	Report any methods used to improve reliability such as restriction of the sample, training of researchers and standardization of methods, and averaging of repeated measurements	n/a
Specific reporting recommendations for studies on measurement error			
ME1	PROM administrations	Report the total number of measurements made and if the measurements were applied to the same samples using the same PROM. The process of administrating the measurements to the patients should be described, including who administered it (i.e., did the patient complete it or was there a proxy), when, how and any time intervals between administrations should be reported. This should include: time interval between repeated measurements (e.g., was the patient stable or not), the test type (e.g., a self-administered questionnaire, an interview-based PROM), the setting in which the instrument was administered (e.g., at the hospital, or at home), and the instructions given for completing it. If relevant, other instruments or measurements accompanying the repeated PROM measurement. Also, if relevant, the independence (whether the PROM was completed without knowledge of the previous completion) of the administrations	n/a
ME2	Statistical analyses	All statistical analyses and results specific to measurement error assessment(s) should be described and their use justified. Specifically, for continuous scores report the Standard Error of Measurement (SEM; Specify the exact model used to calculate the SEM (i.e., SEM consistency or SEM agreement)), Smallest Detectable Change (SDC; specify formula used, included the	n/a

		model of the SEM when based on the SEM) or Limits of Agreement (LoA). For dichotomous/nominal/ordinal scores report marginals (raw data) and the percentage specific (e.g., positive and negative) agreement	
Specific reporting recommendations for studies on criterion validity			
CriV 1	Criterion	Report the details of the criterion used and why it was used. Justification of the gold standard must be reported. Also, describe (if applicable) how and why the criterion was dichotomized or classified. Also, how and when the criterion was administered (e.g., if independent from the PROM)	n/a
CriV 2	Continuous scores	Report correlations (when criterion has continuous scores) or the area under the receiver operating characteristic (ROC) curve (when criterion is dichotomous)	n/a
CriV 3	Categorical scores	Described how (and why) the PROM was dichotomized or made into multiple categories. Report sensitivity and specificity statistics	n/a
Specific reporting recommendations for studies on hypotheses testing for construct validity			
Con V1	Comparator instrument(s)	The comparator instruments should be appropriately described in terms of the construct(s) they intend to measure. Report the measurement properties of the comparator instruments and related citations or data	7
Con V2	Comparator Group(s)	Report characteristics of groups being compared. Include sample sizes in each group	n/a
Con V3	Hypotheses	Report all hypotheses including the direction and magnitude of the expected correlations between the PROM of interest and another measurement instrument, or the direction and magnitude of differences in scores of the PROM between groups	8
Con V4	Statistical analyses	Report all statistical methods and results used to test each hypothesis	8
Con V5	Results	Report which specific results are in accordance with its hypothesis	14
Specific reporting recommendations for studies on responsiveness			
Resp 1	Comparison instrument(s)	The comparator instruments should be appropriately described in terms of the construct(s) they intend to measure. Report the measurement properties of the comparator instruments and related citations or data	n/a
Resp 2	Comparator group(s)	Report characteristics of groups being compared. Include sample sizes in each group	n/a
Resp 3	Hypotheses	Report all hypotheses including the direction and magnitude of the expected correlations between changes in the PROM of interest and change in another measurement instrument, or the direction and magnitude of differences in change scores of the PROM between groups	n/a
Resp 4	Measurement procedures	Report if measurements were applied to the same sample using the same instruments. Describe the measurement procedures, including time intervals between different measurement instruments	n/a
Resp 5	Interim period	The interim period between time points should be described	n/a
Resp 6	Intervention/ exposure	Describe the intervention given or exposure in the interim period if relevant	n/a
Resp 7	Patients changed	Report the proportion of patients that improved or deteriorated (and the details of any anchor used) on the construct measured on all PROMs. Report any changes in scores of the PROM in the target population for the research application relative to the predefined hypotheses	n/a
Resp 8	Statistical analyses	Report all statistical methods and results used to test each hypothesis	n/a
Resp 9	Results	Report which specific results are in accordance with its hypothesis	n/a

Additional file 2: Cognitive Interviews – collaboRATE – Interview Guide.

Needed materials

- Participant consent form (if not previously submitted)
- Demographic questionnaire
- CollaboRATE questionnaire (with translations as closely to the English original) & additional material (translations with simplified sentence structure)
- Interview guide
- **Audio recording device**!

After the interview:

- Incentive of 30 Euros in cash in an envelope
- Form to confirm that the money has been received

Beginning of the interview:

Thank you for agreeing to take part in this interview. This study is about your assessment of the comprehensibility of the German translation of the collaboRATE questionnaire. The aim of the questionnaire is to find out what content was discussed in a consultation with a doctor or therapist. It is already being used successfully in America and some European countries. We are currently looking at whether the German version of the questionnaire is easy to understand.

The interview will take about 30-45 minutes. Everything you say here will be evaluated anonymously. This means that no data will be collected that could be used to identify you personally. For our evaluation, we will record the interview using this audio device. Do you agree to this?

If you wish, you can stop the interview at any time without any disadvantages for you.

During the interview, we will go through the individual questions one by one and I will ask you to rephrase individual sentences or I will ask you questions about them. You are welcome to say out loud anything that is on your mind and "think out loud", so to speak.

During the interview, please imagine that you have just had an appointment with your doctor or therapist and will receive this questionnaire afterwards.

It is important that you keep in mind that this interview is not about whether you think the questionnaire is good or bad, but only about whether you understand the content. This is now the collaboRATE questionnaire [participant receives questionnaire]. Before we take a closer look at the individual aspects together, I would like to give you a little time to look at the questionnaire at your leisure.

We will now go through the questionnaire question by question together.

collaboRATE™ Questionnaire

Is this title understandable? What would make it easier to understand?

Framing sentences:

I: Wenn Sie an den Termin denken, den Sie gerade hatten… [in English: Thinking about the appointment you have just had…]
Is this sentence comprehensible?

IIa: Wenn Sie an den Besuch bei Ihrem Arzt oder Therapeuten heute denken… [in English: Thinking about the visit you had with your physician or health care provider today…]
Is this sentence comprehensible?

I additionally brought an alternative version *[Participant is shown version IIb as a print out]:*

IIb: Wenn Sie an den Besuch bei Ihrem Arzt oder Therapeuten/Ihrer Ärztin oder Therapeutin heute denken… [Explanation: This version verbally differentiates between genders of physicians/healthcare providers.]
Is this sentence more easily comprehensible than the prior one (IIa)?

Wenn Sie an das Gespräch heute mit Ihrem [Arzt oder Therapeut einfügen] über [gesundheitliches Anliegen einfügen] denken… [In English: Thinking about the conversation you had with your [insert physician or health care provider] today about [insert health issue]…]
Is this sentence comprehensible?

Items:

1A: Wie viel Mühe wurde sich gegeben, Ihnen dabei zu helfen, Ihre gesundheitlichen Belange zu verstehen? [in English: How much effort was made to help you understand your health issues?]
Please rephrase the question in your own words.
Who is supposed to have made an effort?
Can you tell me in your own words what you understand as "health issues"?

0	1	2	3	4	5	6	7	8	9
Es wurde sich keine Mühe gegeben. [in English: No effort was made.]									Es wurde sich alle Mühe gegeben. [in English: Every Effort was made.]

How do you understand this scale?
Which end is the most positive, which the most negative?
Can you tell me in your own words what "make no effort" and "make every effort" refer to in relation to the above question?

I additionally brought an alternative version *[Participant is shown version 1B as a printout]:*

1B: Wie sehr wurde Ihnen geholfen, Ihre gesundheitlichen Belange zu verstehen? [In English: How much were you helped to understand your health concerns?]
Is this sentence more easily comprehensible than the prior one (1A)?

2A: Wie viel Mühe wurde sich gegeben, Ihnen zuzuhören, was für Sie bezüglich Ihrer gesundheitlichen Belange am wichtigsten ist? [In English: How much effort was made to listen to the things that matter most to you about your health issues?]
Please rephrase the question in your own words.
What is to be assessed in this question?
Who is the listener in this question?

0	1	2	3	4	5	6	7	8	9
Es wurde sich keine Mühe gegeben. [in English: No effort was made.]									Es wurde sich alle Mühe gegeben. [in English: Every Effort was made.]

Can you tell me in your own words what "make no effort" and "make every effort" refer to in relation to the above question?

I additionally brought an alternative version *[Participant is shown version 2B as a printout]:*

2B: Bezüglich Ihrer gesundheitlichen Belange: Wie sehr wurde Ihnen zugehört, was für Sie am wichtigsten ist? [in English: Regarding your health concerns, how much were you listened to about what is most important to you?]
Is this sentence more easily comprehensible than the prior one (2A)?

3A: Wie viel Mühe wurde sich gegeben, bei der Überlegung, was als nächstes gemacht wird, einzubeziehen, was für Sie am wichtigsten ist? [in English: How much effort was made to include what matters most to you in choosing what to do next?]
Please rephrase the question in your own words.
What is the choice about in this question?
What does "what to do next" refer to?
What is to be included?

0	1	2	3	4	5	6	7	8	9
Es wurde sich keine Mühe gegeben. [in English: No effort was made.]									Es wurde sich alle Mühe gegeben. [in English: Every effort was made.]

Can you tell me in your own words what "make no effort" and "make every effort" refer to in relation to the above question?

I additionally brought an alternative version *[Participant is shown version 3B as a printout]:*

3B: Bei der Überlegung, was als nächstes gemacht wird: Wie sehr wurde einbezogen, was für Sie am wichtigsten ist? [in English: When considering what to do next: How much has what is most important to you been included?]
Is this sentence more easily comprehensible than the prior one (3A)?

This work is licensed under a Creative Commons Attribution 2.0 License.

Additional file 3. Stratification of participants of cognitive interviews.

	n (%)					
	Planned (N=20)		*Invited (N=20)*		*Participated (N=18)*	
**Age**						
< 31 years	5	(25.0)	5	(25.0)	5	(27.8)
31 to 60 years	8	(40.0)	12	(60.0)	10	(55.6)
> 60 years	7	(35.0)	3	(15.0)	3	(16.7)
**Gender**						
Female	10	(50.0)	12	(60.0)	11	(64.7)
Male	10	(50.0)	8	(40.0)	6	(35.3)
**Formal education (highest degree)**						
Graduation after a maximum of 11 years at school	2	(10.0)	2	(10.0)	2	(11.1)
Graduation after more than 11 years at school	4	(20.0)	5	(25.0)	5	(27.8)
Apprenticeship/vocational training	8	(40.0)	6	(30.0)	6	(33.3)
University degree	6	(30.0)	6	(30.0)	5	(27.8)

Notes. Frequencies not adding up to the total number of participants within groups indicate missing data; percentages are calculated for valid data within the group.

collaboRATE™ Fragebogen

(deutsche Version) *

Wenn Sie an den Termin denken, den Sie gerade hatten… **

1. Wie sehr wurde Ihnen geholfen, dass Sie Ihre gesundheitlichen Belange verstehen?

0	1	2	3	4	5	6	7	8	9
überhaupt nicht									sehr

2. Bezüglich Ihrer gesundheitlichen Belange: Wie sehr wurde Ihnen zugehört, was für Sie am wichtigsten ist?

0	1	2	3	4	5	6	7	8	9
überhaupt nicht									sehr

3. Bei der Überlegung, was als nächstes gemacht wird: Wie sehr wurde einbezogen, was für Sie am wichtigsten ist?

0	1	2	3	4	5	6	7	8	9
überhaupt nicht									sehr

Dieses Messinstrument ist geschützt durch eine Creative Commons Attribution 2.0 Lizenz.

_ _

* Bitte verwenden Sie im Deutschen als Titel „Fragebogen zur Zusammenarbeit“ bzw. „Fragen zur Zusammenarbeit“. „collaboRATE“ wurde von deutschsprachigen Personen nicht ausreichend gut verstanden.

** Alternative Einleitungssätze sind:

Wenn Sie an den Besuch bei Ihrem Arzt oder Therapeuten heute denken…

Wenn Sie an den Besuch bei Ihrem Arzt oder Therapeuten/Ihrer Ärztin oder Therapeutin heute denken…

Wenn Sie an das Gespräch heute mit Ihrem [Arzt oder Therapeut einfügen] über [gesundheitliches Anliegen einfügen] denken…

Referenz:

Hahlweg P, Zeh S, Scholl I, Zill J, Dirmaier J, Barr PJ, Elwyn G, Härter M. Translation and psychometric evaluation in cancer care of the German version of collaboRATE™ – a 3-item patient-reported measure of shared decision-making. Health Expectations, 2025.

Additional file 5. Demographic characteristics of the main sample and data sub-sets used for sensitivity analyses.

	Complete sample	Partial samples				
		**Control condition only**	**Intervention condition only**	**Dept. of oncology only**	**Dept. of maxillofacial surgery only**	**Dept. of gynecology only**
**Sample size**, *n*	1,703	819	881	768	325	607
**Age** in years, *mean (SD) [min; max]*	58.0 (15.2) [18; 97]	57.5 (15.2) [18; 94]	58.6 (15.2) [18; 97]	57.3 (15.2) [18; 97]	65.5 (12.0) [30; 91]	54.9 (15.4) [20; 94]
**Gender**, *n (%)*						
Female	1,027 (60.9)	593 (73.1)	432 (49.5)	282 (37.4)	150 (46.3)	593 (98.2)
Male	658 (39.0)	217 (26.8)	440 (50.0)	473 (62.6)	174 (53.7)	10 (1.7)
Other or not specified	1 (0.1)	1 (0.1)	0 (0.0)	0 (0.0)	0 (0.0)	1 (0.2)
**Formal education**, *n (%)*						
Low [a]	295 (17.7)	137 (17.1)	158 (18.4)	127 (17.0)	99 (30.7)	69 (11.6)
Intermediate [b]	518 (31.1)	244 (30.4)	272 (31.6)	208 (27.8)	120 (37.2)	188 (31.7)
High [c]	381 (22.9)	194 (24.2)	186 (21.6)	178 (23.8)	50 (15.5)	152 (25.6)
Very high [d]	458 (27.5)	220 (27.4)	238 (27.6)	227 (30.4)	51 (15.8)	180 (30.4)
Other	14 (0.8)	7 (0.9)	7 (0.8)	7 (0.9)	3 (0.9)	4 (0.7)
**Occupational status** [e], *n (%)*						
Retired	756 (45.1)	369 (45.7)	386 (44.6)	326 (43.5)	202 (62.5)	227 (37.8)
(Self-)employed	741 (44.2)	355 (44.0)	484 (44.3)	333 (44.4)	99 (30.7)	307 (51.2)
Homemaker	(< 5.0)	42 (5.2)	(< 5.0)	(< 5.0)	20 (6.2)	(< 5.0)
Other [f] (< 5% each)	255 (15.2)	82 (10.1)	131 (15.1)	100 (13.3)	25 (7.7)	87 (14.3)
**Mother tongue**, *n (%)*						
German	1525 (92.9)	732 (92.7)	791 (93.1)	689 (94.1)	305 (95.6)	529 (89.8)
Other	107 (6.5)	55 (7.0)	52 (6.1)	41 (5.6)	11 (3.4)	55 (9.3)
Bilingual (German and other)	10 (0.6)	3 (0.4)	7 (0.8)	2 (0.3)	3 (0.9)	5 (0.8)
**Setting**, *n (%)*						
Outpatient	1548 (91.1)	745 (91.1)	802 (91.0)	688 (89.6)	274 (84.6)	585 (96.4)
Inpatient	152 (8.9)	73 (8.9)	79 (9.0)	80 (10.4)	50 (15.4)	22 (3.6)
**Diagnosis**, *n (%)*						
Confirmed malignant neoplasm	1,588 (93.2)	759 (92.7)	826 (93.8)	722 (94.0)	318 (97.8)	545 (89.8)
Suspected malignant neoplasm	20 (1.2)	11 (1.3)	9 (1.0)	3 (0.4)	3 (0.9)	14 (2.3)
In-situ neoplasm or neoplasm of uncertain behavior	54 (3.2)	19 (2.3)	35 (4.0)	37 (4.8)	3 (0.9)	14 (2.3)
Benign neoplasm	41 (2.4)	30 (3.6)	11 (1.2)	6 (0.8)	1 (0.3)	34 (5.6)

**Time since initial diagnosis**, *n (%)*												
less than 1 year	671	(44.5)	338	(47.4)	331	(42.6)	281	(39.9)	110	(37.4)	278	(54.7)
1 to 5 years	567	(37.6)	244	(34.2)	323	(40.7)	277	(39.3)	151	(51.4)	139	(27.4)
more than 5 years	271	(18.0)	131	(18.4)	139	(17.5)	146	(20.7)	33	(11.2)	91	(17.9)
**Reason for visit** [e g], *n (%)*												
Diagnostic investigation	301	(17.9)	158	(19.5)	142	(16.3)	106	(14.0)	33	(10.3)	161	(26.7)
Initial communication of the diagnosis	270	(16.1)	141	(17.6)	127	(14.7)	93	(12.4)	64	(20.0)	112	(18.6)
Treatment planning	437	(21.1)	233	(28.9)	204	(23.6)	132	(17.6)	110	(34.4)	195	(32.4)
Treatment	347	(20.7)	138	(17.1)	208	(24.1)	181	(24.1)	83	(25.9)	82	(13.6)
Treatment monitoring	503	(30.0)	216	(26.8)	286	(33.1)	307	(40.9)	40	(12.5)	155	(25.8)
Aftercare	364	(21.7)	178	(22.1)	184	(21.3)	112	(14.9)	152	(47.5)	98	(16.3)
**Decision topic** [e g], *n (%)*												
Diagnostic procedures	474	(29.5)	241	(31.1)	233	(28.1)	207	(28.4)	65	(21.9)	202	(34.9)
Surgery	454	(28.3)	250	(32.3)	204	(24.6)	71	(9.7)	184	(62.0)	199	(34.4)
Chemotherapy	527	(32.8)	186	(24.0)	339	(40.8)	394	(54.0)		(< 5.0)	120	(20.8)
Radiation therapy	149	(9.3)	60	(7.7)	88	(10.6)	72	(9.9)	33	(11.1)	43	(7.4)
Other treatment	169	(10.5)	90	(11.6)	79	(9.5)	85	(11.6)		(< 5.0)	70	(12.1)
Unclear, if decision was made		(< 5.0)		(< 5.0)		(< 5.0)		(< 5.0)	17	(5.7)		(< 5.0)
**Overall health**, *mean (SD) [min; max]*	3.2	(0.8)	3.2	(0.8)	3.2	(0.8)	3.3	(0.8)	3.2	(0.8)	3.1	(0.9)
1 to 5, higher better		[1; 5]		[1; 5]		[1; 5]		[1; 5]		[1; 5]		[1; 5]
**Distress**, *mean (SD) [min; max]*	5.6	(2.4)	5.7	(2.5)	5.5	(2.4)	5.5	(2.4)	5.2	(2.4)	5.8	(2.5)
0 to 10, higher more		[0; 10]		[0; 10]		[0; 10]		[0; 10]		[0; 10]		[0; 10]
**SDM-Q-9 sum score**, *mean (SD) [min; max]*	64.4	(26.5)	64.1	(26.4)	64.9	(26.6)	64.8	(26.0)	62.0	(28.7)	65.3	(25.9)
1 to 100, higher more SDM		[0; 100]		[0; 100]		[0; 100]		[0; 100]		[0; 100]		[0; 100]
**Patient satisfaction**, *mean (SD) [min; max]*	3.5	(0.8)	3.4	(0.9)	3.5	(0.7)	3.5	(0.7)	3.4	(0.9)	3.4	(0.9)
1 to 4, higher more		[1; 4]		[1; 4]		[1; 4]		[1; 4]		[1; 4]		[1; 4]

Notes. Frequencies not adding up to the total number of participants within groups indicate missing data; percentages are calculated for valid data within the group; SD=standard deviation; Dept.=department; [a] low = no formal degree or graduation after less than 10 years at school; [b] intermediate = graduation after 10 or 11 years at school; [c] high = graduation after more than 11 years at school; [d] very high = college or university degree; [e] multiple choices possible; [f] including homemaker (if not separately displayed), student/trainee, sick leave, parental leave, military service, unemployed; [g] only categories with more than five percent of the total sample are displayed.

Additional file 6. Response distributions of the German collaboRATE items.

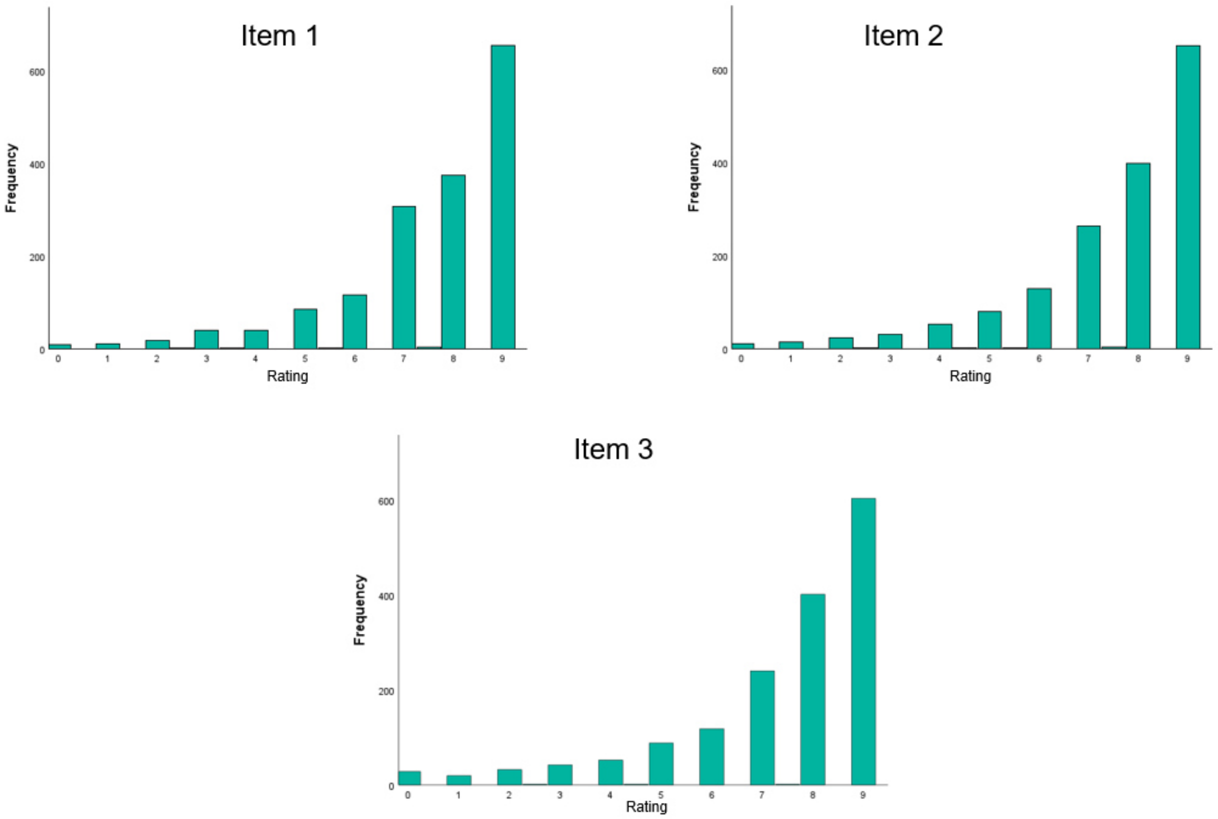

<u>Legend:</u>
Item 1 = How much were you helped to understand your health issues?
Item 2 = Regarding your health issues: How much were you listened to about what matters most to you?
Item 3 = When choosing what to do next: How much has been considered what matters most to you?
0 = not at all
9 = very much

Additional file 7. Item characteristics in the main sample and data sub-sets used for sensitivity analyses.

Item	n_{valid}	Mean	SD	[min; max]	Skew-ness	Item diffi-culty	Com-pletion rate, %	"not at all", % [a]	"very much", % [a]
**Complete Sample**									
Item 1	1,658	7.54	1.79	[0; 9]	-1.64	0.84	97.4	0.5	39.4
Item 2	1,655	7.51	1.84	[0; 9]	-1.65	0.83	97.2	0.6	39.3
Item 3	1,626	7.32	2.10	[0; 9]	-1.63	0.81	95.5	1.7	37.1
**Partial sample: Control condition**									
Item 1	795	7.54	1.81	[0; 9]	-1.57	0.84	97.1	0.5	41.8
Item 2	797	7.50	1.89	[0; 9]	-1.59	0.83	97.3	0.6	41.8
Item 3	780	7.29	2.20	[0; 9]	-1.62	0.81	95.2	2.3	39.0
**Partial sample: Intervention condition**									
Item 1	860	7.53	1.75	[0; 9]	-1.71	0.84	97.6	0.6	37.3
Item 2	855	7.52	1.79	[0; 9]	-1.71	0.84	97.0	0.6	37.0
Item 3	843	7.34	1.98	[0; 9]	-1.61	0.82	96.0	1.1	35.0
**Partial sample: Department of Oncology**									
Item 1	745	7.46	1.76	[0; 9]	-1.58	0.83	97.0	0.5	35.2
Item 2	747	7.45	1.81	[0; 9]	-1.59	0.83	97.3	0.5	35.6
Item 3	732	7.22	2.08	[0; 9]	-1.59	0.80	95.3	1.6	32.7
**Partial sample: Department of Maxillofacial Surgery**									
Item 1	318	7.56	1.90	[0; 9]	-1.93	0.84	97.8	1.3	41.2
Item 2	315	7.40	1.99	[0; 9]	-1.73	0.82	96.9	1.6	37.8
Item 3	312	7.25	2.21	[0; 9]	-1.63	0.81	96.0	2.6	37.2
**Partial sample: Department of Gynecology**									
Item 1	592	7.64	1.73	[0; 9]	-1.52	0.85	97.5	0.2	43.9
Item 2	590	7.66	1.79	[0; 9]	-1.67	0.85	97.2	0.2	44.7
Item 3	579	7.47	2.03	[0; 9]	-1.66	0.83	95.4	1.2	42.5
**Partial sample: Age ≤ 58 years**									
Item 1	811	7.49	1.74	[0; 9]	-1.52	0.83	99.3	0.5	35.6
Item 2	806	7.42	1.82	[0; 9]	-1.47	0.82	98.7	0.4	35.4
Item 3	799	7.19	2.08	[0; 9]	-1.48	0.80	97.8	1.4	31.8
**Partial sample: Age > 58 years**									
Item 1	826	7.58	1.82	[0; 9]	-1.75	0.84	97.3	0.6	42.6
Item 2	828	7.59	1.85	[0; 9]	-1.82	0.84	97.5	0.8	42.5
Item 3	806	7.44	2.11	[0; 9]	-1.80	0.84	95.0	2.1	42.1
**Partial sample: Gender = female**									
Item 1	1,006	7.51	1.78	[0; 9]	-1.47	0.83	98.0	0.2	39.9
Item 2	1,002	7.48	1.88	[0; 9]	-1.61	0.83	97.6	0.6	39.6
Item 3	987	7.29	2.13	[0; 9]	-1.54	0.81	96.1	1.5	37.6
**Partial sample: Gender = male**									
Item 1	648	7.58	1.79	[0; 9]	-1.90	0.84	98.5	1.1	38.9
Item 2	649	7.55	1.78	[0; 9]	-1.70	0.84	98.6	0.6	38.8
Item 3	636	7.36	2.05	[0; 9]	-1.77	0.82	96.7	2.0	36.3
**Partial sample: Education level = low**									
Item 1	289	7.69	1.69	[0; 9]	-1.82	0.85	98.0	0.3	43.3
Item 2	289	7.74	1.71	[0; 9]	-2.05	0.86	98.0	0.7	43.6
Item 3	280	7.41	2.24	[0; 9]	-1.82	0.82	94.9	3.6	43.2
**Partial sample: Education level = intermediate**									
Item 1	510	7.57	1.80	[0; 9]	-1.76	0.84	98.5	0.8	39.6
Item 2	512	7.56	1.85	[0; 9]	-1.82	0.84	98.8	0.8	40.0
Item 3	503	7.44	2.03	[0; 9]	-1.81	0.83	97.1	1.6	39.2

**Partial sample: Education level = high**									
Item 1	372	7.54	1.70	[0; 9]	-1.39	0.84	97.6	0.8	39.0
Item 2	370	7.47	1.47	[0; 9]	-1.29	0.83	97.1	0.5	40.0
Item 3	366	7.33	1.94	[0; 9]	-1.47	0.81	96.1	0.5	34.2
**Partial sample: Education level = very high**									
Item 1	453	7.40	1.90	[0; 9]	-1.60	0.82	98.9	0.9	36.9
Item 2	450	7.34	1.95	[0; 9]	-1.54	0.82	98.3	0.9	34.9
Item 3	445	7.10	2.20	[0; 9]	-1.42	0.80	97.2	1.8	32.8
**Partial sample: Setting = outpatient**									
Item 1	1,506	7.54	1.76	[0; 9]	-1.62	0.84	97.3	0.5	38.8
Item 2	1,501	7.51	1.84	[0; 9]	-1.63	0.83	97.0	0.5	38.9
Item 3	1,477	7.35	2.05	[0; 9]	-1.64	0.82	95.4	1.5	37.1
**Partial sample: Setting = inpatient**									
Item 1	149	7.61	1.94	[0; 9]	-1.87	0.85	98.0	1.3	47.0
Item 2	151	7.59	1.87	[0; 9]	-1.83	0.84	99.3	1.3	43.7
Item 3	146	7.00	2.40	[0; 9]	-1.42	0.78	96.1	3.4	37.0

Notes. All items were rated on a 10-point anchored scale ranging from 0 (“not at all”) to 9 (“very much”); SD=standard deviation; Item 1 = “How much were you helped to understand your health issues?”; Item 2 = “Regarding your health issues: How much were you listened to about what matters most to you?”; Item 3 = “When choosing what to do next: How much has been considered what matters most to you?”; [a] percentages were calculated for valid data for this item.

Additional file 8. Convergent and divergent validity in the main sample and data sub-sets used for sensitivity analyses.

		Partial samples				
	**Complete sample**	**Control condition only**	**Inter-vention condition only**	**Dept. of oncology only**	**Dept. of maxilla-facial surgery only**	**Dept. of gyne-cology only**
**Sample size**, *n*	1,703	819	881	768	325	607
**Convergent validity** between SDM-Q-9 and… (hypothesis > 0.7), *n*						
… collaboRATE sum score, *r*	0.47**	0.47**	0.46**	0.46**	0.46**	0.48**
… collaboRATE top score, *pbr*	0.27**	0.27**	0.26**	0.29**	0.20**	0.28**
n	1,429	688	738	644	265	517
**Convergent validity** of patient satisfaction with care and… (hypothesis = 0.5), *n*						
… collaboRATE sum score, r_s	0.46**	0.42**	0.50**	0.51**	0.41**	0.45**
… collaboRATE top score, *Cramer's V (Chi^2)*	0.37** (218.30)	0.40** (120.18)	0.36** (106.04)	0.37** (97.97)	0.36** (40.77)	0.38** (82.10)
n	1,590	762	825	717	307	563
**Divergent validity** of patient distress and… (hypothesis = 0.1) , *n*						
… collaboRATE sum score, *r*	-0.11**	-0.12**	-0.10**	-0.11**	-0.19**	-0.07
… collaboRATE top score, *pbr*	-0.09**	-0.09*	-0.09*	-0.09*	-0.17**	-0.05
n	1,586	762	821	712	304	567

Notes. n: sample size; r: Pearson Product Moment Correlation; pbr: point-biserial correlation; r_s: Spearman rank correlation; ** p<.01; * p<.05.
